# Species identification of livefood flightless fly (*Torinido‐shoujoubae*) through DNA barcoding

**DOI:** 10.1002/ece3.11622

**Published:** 2024-07-08

**Authors:** Koh Nakagawa, Kaoru Ogino, Takehiro K. Katoh, Nobuaki Kono

**Affiliations:** ^1^ Institute for Advanced Biosciences Keio University Tsuruoka Yamagata Japan; ^2^ Faculty of Environment and Information Studies Keio University Fujisawa Kanagawa Japan; ^3^ Department of Biological Sciences Tokyo Metropolitan University Hachioji Tokyo Japan; ^4^ Graduate School of Media and Governance Keio University Fujisawa Kanagawa Japan

**Keywords:** DNA barcoding, *Drosophila hydei*, flightless fly, insect feed

## Abstract

*Torinido‐shoujoubae,* as it is called in Japanese, is a flightless *Drosophila* sp. that is sold commercially in Japan. This *Drosophila* sp. is often used as feeds for model organisms such as reptiles and spiders. There is no scientific name provided for the fruit fly that is known as *Torinido‐shoujoubae*, as well as any historical background or data behind this species. There has been a previous study that was conducted through morphological characteristics analysis of the body as well as the male copulatory organ and has been estimated as *Drosophila hydei.* The objective of this study was to determine the species of this unidentified fly known as *Torinido‐shoujoubae* based on a molecular evidence with a DNA barcoding. Samples were purchased from four separate suppliers to examine whether there are any differences between them. COI regions were amplified using PCR and the sequenced results were aligned against two databases, NCBI and BOLD. *Torinido‐shoujoubae* samples provided from all suppliers were confirmed to be *D. hydei*.

## Graphical abstract

*Torinido‐shoujoubae*, a flightless *Drosophila* sp. sold commercially, is often used as feed for model organisms like reptiles and spiders. This study used DNA barcoding to determine that *Torinido‐shoujoubae* samples from four different suppliers were all identified as *Drosophila hydei*.

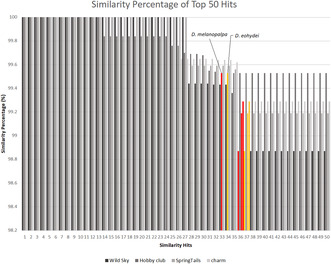

## 1 INTRODUCTION

Flightless fly models are valuable in a range of experimental settings, serving as a model organism to function as a live feed. As a research model, these flightless flies are invaluable in studies of neuromuscular diseases such as spinal muscular atrophy, spinobulbar muscular atrophy, myotonic dystrophy, and dystrophinopathies (Lloyd & Taylor, 2010; Rajendra et al., 2007). In practical applications, flightless *Drosophila* is used as live feeds for pet reptiles and amphibians, ensuring easier management owing to its flightlessness and inability to escape enclosure (Lourenço et al., 2022; Michalska et al., 2023; Roberts‐McEwen et al., 2022). Flightless fruit flies (Order: Diptera) encompass a variety of different species of flies, including *Drosophila melanogaster*, *Drosophila hydei*, *Bactrocera dorsalis* and *Bactocera cucurbitae*, which has mutations on genes such as the SNM genes, or disruptions in central nervous pathways from pyrethroid poisoning that cause them to be flightless (Lloyd & Taylor, 2010; McCombs & Saul, 1992; Michalska et al., 2023; Roberts‐McEwen et al., 2022; Zheng et al., 2019).

In Japan, when conducting experiments involving flightless flies or as live feeds, two types of flightless flies are available from stores. One is the wingless mutant of the common fruit fly, scientifically named, *D. melanogaster* and the other is the flightless *Torinido*‐*shoujoubae*. The precise species name of *Torinido*‐*shoujoubae* is not specified by the stores and is simply recorded as *Drosophila* sp.

In a previous study, the features of the whole body were mentioned as, “It falls within the larger sized group of the *Drosophila*, having a dark body color and a thoracic dorsum with mottled pattern. The banded patterns (black bands) on each abdominal tergite cuts off in the center while protruding towards the head on both ends” and the characteristics of the reproductive organs noted as, “the male copulatory organ had a hook‐like structure unique to aedeagus”, to conduct taxonomic diagnosis that *Torinido*‐*shoujoubae* was *D*. *hydei* (*Kasuri*‐*shoujoubae* in Japanese).

*Drosophila hydei* is a model organism used in genetics and molecular biology (Akhmanova et al., 1995; Ryder & Russell, 2003; Syomin et al., 2001), as well as, in ecological studies (Markow, 2015; Yang, 2018) and phylogenetic studies (Morán & Fontdevila, 2005). In genetics, *D*. *hydei* is used for comparative studies of gene functions with representative models such as *D*. *melanogaster* and other *Drosophila* species (Akhmanova et al., 1995; Templeton et al., 1989) and for genetic studies based on *D*. *hydei* as a model organism (Hennig, 1985; Neesen et al., 1994). Additionally, in ecological studies, interactions with symbiotic bacteria (Gerth et al., 2021; Griffin et al., 2022; Kageyama et al., 2006), roles as mediators of yeast (Lam & Howell, 2015) and parasitic relationship with mites and parasitoid wasps (Corbin et al., 2021; Michalska et al., 2023; Perez‐Leanos et al., 2017) have been studied. Furthermore, *D*. *hydei*, as one of the flightless flies, is preferred by researchers for use in laboratories as feeds for predating species such as spiders and beetles (Takasuka & Arakawa, 2023) due to its ease of maintenance and provision. Likewise, it has a larger body size in contrast to *D*. *melanogaster* (Markow, 2015), which is expected to provide adequate nutritional content to the experimental species.

As such, *D*. *hydei* is valuable as a source for various biological studies and as live feeds for experimental animals. Therefore, establishing a global standard for experiments using *D*. *hydei* is crucial. In order to achieve this, it is necessary to conduct species identification not only based on morphological characteristics but also through DNA barcoding to ensure the reproducibility of experiments regardless of the experimental model. It has been previously pointed out that diagnosis through morphological identification does not always correspond to species classification and that cryptic species cannot be distinguished (Hebert et al., 2004). There has been an advocacy for transitioning from a morphological species identification to a taxonomic system based on DNA barcoding (Hebert et al., 2003; Jackson & Nijman, 2020). Therefore, we aimed to verify commercially available “*Torinido*‐*shoujoubae*” as *D. hydei* through molecular characteristics.

## 2 MATERIALS AND METHODS

### 2.1 *Torinido‐shoujoubae* samples

The *Torinido‐shoujoubae* samples were purchased from four stores that marketed flightless flies as *Torinido*‐*shoujoubae*. The companies are listed as follows:

1. Hobby club (Aichi, Japan: https://item.rakuten.co.jp/hobbyclub/10001924/?scid=wi_ichi_iphoneapp_item_share [Accessed 27 June 2023].)
2. charm (Gunma, Japan: https://www.shopping‐charm.jp/product/2c2c2c2c‐2c2c‐2c2c‐2c2c‐2c3232313336 [Accessed 27 June 2023])
3. Wild Sky (Tokyo, Japan: https://shop.wildsky.net/item‐detail/328586 [Accessed 27 June 2023])
4. SpringTails (Ibaraki, Japan: https://springtails.ocnk.net/product/8 [Accessed 27 June 2023]).

### 2.2 DNA extraction

The fly samples were anesthetized by carbon dioxide. Legs were removed carefully using disinfected tweezers and placed within the Biomasher II tube. A voume of 50 μL of Lysis buffer [20 mM Tris–HCl, 100 mM NaCl, 5 mM EDTA, and 0.1% SDS] was added into the Biomasher II tube, followed by the grinding of the leg samples. A voume of 1 μL of Proteinase K was added and left to incubate at 60°C for 5 min, followed by 98°C for 2 min, and finally, at room temperature for 10 min. The incubated solution was then placed in a centrifuge to separate the DNA from the other contaminants and materials, such as the fragments of the ground limbs of the flightless *Drosophila* sp. The supernatant of the solution which contains the DNA was transferred to a new 0.2 mL tube for PCR.

### 2.3 PCR

The DNA barcoding was implemented according to the methods of the previous study (Iwai et al., 2021). The cytochrome oxidase subunit I (COI) genes were amplified using PCR with appropriate primers (Table 1; Folmer et al., 1994; Hedin & Maddison, 2001; Simon et al., 1994) according to the recommended conditions, 94°C, (98°C 10 s, 55°C 15 s, 68°C 1 min) × 30, 68°C 1 min. The purification and quantification procedures for PCR product were conducted with the use of AMPure beads and a Fluorometer measurement, respectively. The Fluorometer measurements were taken using the Qubit 3 Fluorometer along with the dsDNA HS (High Sensitivity) Working Solution. PCR products were observed with agarose gel electrophoresis using a 1% gel. Referring to Table 1, DNA fragments amplified using set A were expected to appear within the range around 725 bp, as compared to the DNA marker placed alongside the samples. Set B, on the other hand, was expected to appear in the region around 1082 bp, which is its fragment size. Successful amplification of the DNA was confirmed if the bands appeared at 725 bp and 1082 bp regions for set A and set B, respectively.

**TABLE 1:** Set A and B of COI primers of overlapping regions.

Set	Primers	Sequence	Amplicon length
A	COI1490	5′‐GGTCAACAAATCATAAAGATATTGG‐3′	725
	COI2198	5′‐TAAACTTCAGGGTGACCAAAAAATCA‐3′	
B	COI1718	5′‐AGGATAGTCAGAAACAAAGCTAAGC‐3′	1082
	COI2776	5′‐CGTGCAGCCTGAGAAGAAGT‐3′	

### 2.4 Sequencing and bioinformatic analysis

The PCR products were sequenced by Eurofins Genomics (Tokyo, Japan). The sequenced data from primer set A and B were aligned together with a MAFFT (Katoh et al., 2019). The aligned sequences can be, then, run through BLAST on two databases: the National Center for Biotechnology Information (NCBI) and the Barcode of Life Database (BOLD) (Altschul et al., 1990; Ratnasingham & Hebert, 2007). IQ‐TREE 2.2.5 (Minh et al., 2020) was utilized in order to construct the phylogenetic tree based on the COI regions from the ranging flightless fruit fly species that were aligned using MAFFT. The phylogenetic tree includes a bootstrap value that was calculated using 1000 replicates. The COI sequences of each known species were gathered from the BOLD database: *D*. *eohydei* (GBDP2608‐06), *D*. *hydei* (ASDMT1779‐11), *D*. *melanogaster* (ACLB002‐06), *D*. *sechellia* (GBDP2869‐06). The tree was rooted using the midpoint method to ensure the balanced representation of the known species and the target unknown fruit fly. The phylogenetic tree was visualized and edited using a software called Figtree v1.4.4 (http://tree.bio.ed.ac.uk/software/figtree/ [Accessed 4 December 2023]).

### 2.5 Morphological examination

The flies were fixed with 70% ethanol, and of which external morphology was examined under an optical stereo microscope (Olympus SZX7). The specimens were dissected to observe detailed structures of the male and female terminalia, wing, and male foreleg. These dissected parts were treated with 10% KOH solution at 85°C for a few minutes and then mounted in a droplet of glycerin on a glass slide to observe under a stereo or light microscope. The specimens and organs were microphotographed using a Dino‐Lite® Microscope Eyepiece Camera (ANMO Electronics Corporation). Cumming and Wood (2017) were followed for morphological terminology.

## 3 RESULTS AND DISCUSSION

This study employs a molecular biological approach for the species identification of the flightless fly that is known as *Torinido‐shoujoubae*. DNA barcoding method, in comparison to the morphological analysis, has a higher replicability and reliability in terms of species identification, where only fragments are required (Chan et al., 2014). This is a molecular technique for species identification that implements a genetic marker of short and uniform DNA sequences within a specified region of the gene (Hebert et al., 2003). Cytochrome c oxidase subunit I (COI) is a genetic marker for DNA barcoding, derived from a mitochondrial gene that is highly conserved within a wide range of species including *Drosophila* (Hebert et al., 2003; Yassin et al., 2010). The COI region used in this study has been specifically designed for arthropods in past works by other researchers (Folmer et al., 1994; Hedin & Maddison, 2001) establishing a foundation for its use in genetic studies across a variety of arthropod species. This region has been widely utilized in research encompassing a diverse array of species, including spiders and ants (Arakawa et al., 2022; Iwai et al., 2021) This consistent application across different studies underscores the COI region's utility and reliability in phylogenetic and species identification research within the arthropod community. Flightless flies from four different stores, all sold under the name *Torinido‐shoujoubae*, were purchased and subjected to detailed examination. The COI sequences produced were deposited in GenBank under the accession numbers PP663876, PP663875, PP663877, and PP663878 corresponding to the *Torinido*‐*shoujoubae* samples from stores Hobby club, charm, SpringTails and Wild Sky respectively. The resulting sequences were compared against existing databases. Morphological species identification was conducted alongside DNA barcoding to further support the results.

**FIGURE 1:**
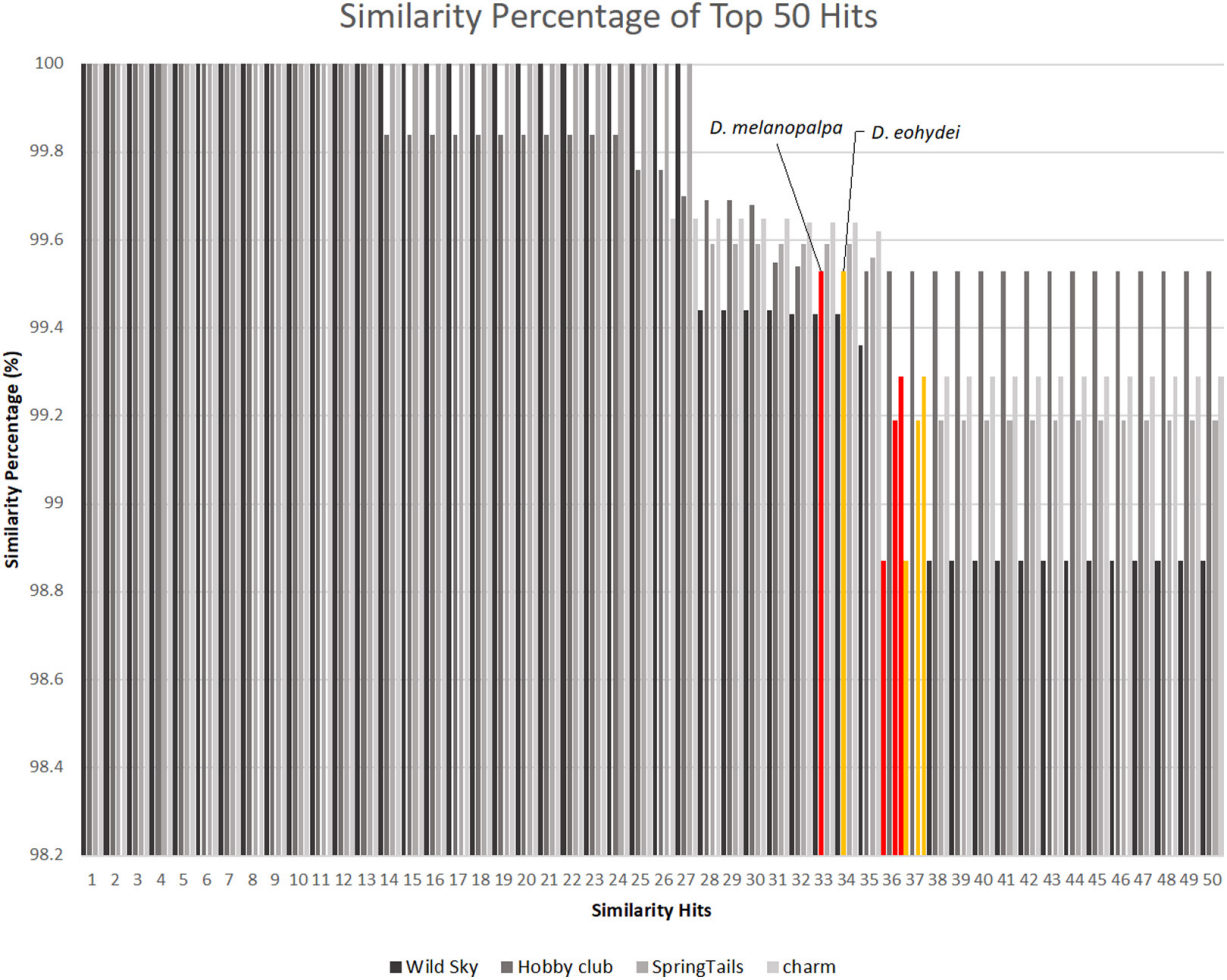
Bar graph of the top 50 BLAST Results on BOLD. The gray scale bars represents all the data aligned with *Drosophila hydei*. The red and orange bars are the only two outlier species found in all search of the samples from the four companies.

**FIGURE 2:**
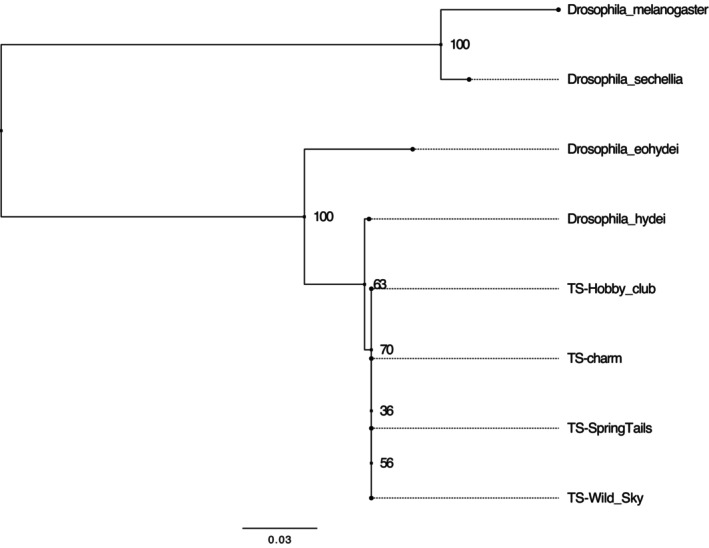
Phylogenetic Tree of *Torinido‐shoujoubae* and known species. TS is an abbreviation for *Torinido‐shoujoubae*. Values located at the node refers to the bootstrap values.

The results from the DNA barcoding analysis with NCBI and BOLD presented that all *Torinido‐shoujoubae* from four different stores were all *D. hydei* (Table 2). All the top 50 hits from BOLD were aligned with *D. hydei* at a similarity percentage higher than 98%, excluding only 2 outlier species within each of the samples from the four companies (Figure 1). The two outliers species were found in all samples and are most likely to be a data contamination. The scarcity of outliers and the consistency of data with high similarity percentage underscores the high reliability and consistency of the data to further support that all *Torinido‐shoujoubae* were all *D. hydei*. Through the implementation of IQ‐TREE 2.2.5, the phylogenetic tree was constructed based on COI regions as shown in Figure 2. The tree reiterates the results from the BLAST search that the unknown fruit fly is most closely related to *D*. *hydei* with the branch length of 0.0027 between the two. Although there is a slight diversion between *D*. *hydei* and the given samples, possibly due to the shorter sequenced data, the results are confidently supported with the BLAST search on two different databases stating that it is *D*. *hydei*.

**TABLE 2:** Top 10 hits based on similarity percentage from the samples of four stores.

Company	Description	*Genus*	*Species*	Similarity percentage	Accession
Charm	*Drosophila hydei* isolate KM009 cytochrome c oxidase subunit I (COI) gene, partial cds; mitochondrial	*Drosophila*	*hydei*	100.00%	OQ058868.1
	*Drosophila hydei* isolate KM007 cytochrome c oxidase subunit I (COI) gene, partial cds; mitochondrial	*Drosophila*	*hydei*	100.00%	OQ058867.1
	*Drosophila hydei* voucher DHYDE20161106 mitochondrion, complete genome	*Drosophila*	*hydei*	100.00%	MK659821.1
	*Drosophila hydei* strain 15,085–1641.58 cytochrome c oxidase subunit I (COI) gene, partial cds; mitochondrial	*Drosophila*	*hydei*	100.00%	EU390734.1
	*Drosophila hydei* mitochondrial partial COI gene for cytochrome oxidase subunit 1	*Drosophila*	*hydei*	99.65%	LN867077.1
	*Drosophila hydei* isolate ABH5_carrier_of_Spiroplasma cytochrome c oxidase subunit I (COI) gene, partial cds; mitochondrial	*Drosophila*	*hydei*	99.65%	OK037196.1
	*Drosophila hydei* strain Africa cytochrome oxidase subunit I (COI) gene, partial cds; mitochondrial	*Drosophila*	*hydei*	99.65%	DQ471603.1
	*Drosophila hydei* cytochrome oxidase subunit I (COI) gene, partial cds; mitochondrial	*Drosophila*	*hydei*	99.65%	DQ471602.1
	*Drosophila hydei* isolate J86 cytochrome c oxidase subunit I (COI) gene, partial cds; mitochondrial	*Drosophila*	*hydei*	99.62%	KX275231.1
	*Drosophila hydei* isolate hydei54 cytochrome c oxidase subunit 1 (COI) gene, partial cds; mitochondrial	*Drosophila*	*hydei*	99.29%	MH142773.1
SpringTails	*Drosophila hydei* isolate KM009 cytochrome c oxidase subunit I (COI) gene, partial cds; mitochondrial	*Drosophila*	*hydei*	100.00%	OQ058868.1
	*Drosophila hydei* isolate KM007 cytochrome c oxidase subunit I (COI) gene, partial cds; mitochondrial	*Drosophila*	*hydei*	100.00%	OQ058867.1
	*Drosophila hydei* voucher DHYDE20161106 mitochondrion, complete genome	*Drosophila*	*hydei*	100.00%	MK659821.1
	*Drosophila hydei* strain 15,085–1641.58 cytochrome c oxidase subunit I (COI) gene, partial cds; mitochondrial	*Drosophila*	*hydei*	100.00%	EU390734.1
	*Drosophila hydei* strain Africa cytochrome oxidase subunit I (COI) gene, partial cds; mitochondrial	*Drosophila*	*hydei*	100.00%	DQ471603.1
	*Drosophila hydei* cytochrome oxidase subunit I (COI) gene, partial cds; mitochondrial	*Drosophila*	*hydei*	100.00%	DQ471602.1
	*Drosophila hydei* mitochondrial partial COI gene for cytochrome oxidase subunit 1	*Drosophila*	*hydei*	99.59%	LN867077.1
	*Drosophila hydei* isolate ABH5_carrier_of_Spiroplasma cytochrome c oxidase subunit I (COI) gene, partial cds; mitochondrial	*Drosophila*	*hydei*	99.59%	OK037196.1
	*Drosophila hydei* isolate J86 cytochrome c oxidase subunit I (COI) gene, partial cds; mitochondrial	*Drosophila*	*hydei*	99.56%	KX275231.1
	*Drosophila hydei* isolate hydei54 cytochrome c oxidase subunit 1 (COI) gene, partial cds; mitochondrial	*Drosophila*	*hydei*	99.19%	MH142773.1
Hobby club	*Drosophila hydei* voucher DHYDE20161106 mitochondrion, complete genome	*Drosophila*	*hydei*	99.73%	MK659821.1
	*Drosophila hydei* strain Africa cytochrome oxidase subunit I (COI) gene, partial cds; mitochondrial	*Drosophila*	*hydei*	99.73%	DQ471603.1
	*Drosophila hydei* cytochrome oxidase subunit I (COI) gene, partial cds; mitochondrial	*Drosophila*	*hydei*	99.55%	DQ471602.1
	*Drosophila hydei* voucher 105,429 NADH dehydrogenase subunit 2 (ND2) gene, partial cds; tRNA‐Trp and tRNA‐Cys genes, complete sequence; tRNA‐Tyr gene, partial sequence; and cytochrome c oxidase subunit I (COI) gene, partial cds; mitochondrial	*Drosophila*	*hydei*	99.13%	EU493606.1
	*Drosophila hydei* cytochrome oxidase subunit I (COI) gene, complete cds; mitochondrial	*Drosophila*	*hydei*	99.09%	JQ679112.1
	*Drosophila eohydei* cytochrome oxidase subunit I (COI) gene, partial cds; mitochondrial	*Drosophila*	*eohydei*	94.91%	DQ471601.1
	*Drosophila sechellia* voucher DSECH20161109 mitochondrion, complete genome	*Drosophila*	*sechellia*	88.77%	MK659840.1
	*Drosophila sechellia* mitochondrion, complete genome	*Drosophila*	*sechellia*	88.77%	NC_005780.1
	*Drosophila melanogaster* strain ANU‐1 mitochondrion, complete genome	*Drosophila*	*melanogaster*	88.76%	OR555819.1
	*Drosophila sechellia* mitochondrial cytochrome c oxidase subunit I (COI) gene, Trp‐, Cys‐, and Tyr‐ tRNA genes, NADH dehydrogenase subunit 2 (ND2) gene, 3′ end	*Drosophila*	*sechellia*	88.68%	M57908.1
Wildsky	*Drosophila hydei* isolate KM009 cytochrome c oxidase subunit I (COI) gene, partial cds; mitochondrial	*Drosophila*	*hydei*	100.00%	OQ058868.1
	*Drosophila hydei* isolate KM007 cytochrome c oxidase subunit I (COI) gene, partial cds; mitochondrial	*Drosophila*	*hydei*	100.00%	OQ058867.1
	*Drosophila hydei* voucher DHYDE20161106 mitochondrion, complete genome	*Drosophila*	*hydei*	100.00%	MK659821.1
	*Drosophila hydei* strain 15,085–1641.58 cytochrome c oxidase subunit I (COI) gene, partial cds; mitochondrial	*Drosophila*	*hydei*	100.00%	EU390734.1
	*Drosophila hydei* strain Africa cytochrome oxidase subunit I (COI) gene, partial cds; mitochondrial	*Drosophila*	*hydei*	100.00%	DQ471603.1
	*Drosophila hydei* cytochrome oxidase subunit I (COI) gene, partial cds; mitochondrial	*Drosophila*	*hydei*	100.00%	DQ471602.1
	*Drosophila hydei* mitochondrial partial COI gene for cytochrome oxidase subunit 1	*Drosophila*	*hydei*	99.45%	LN867077.1
	*Drosophila hydei* isolate ABH5_carrier_of_Spiroplasma cytochrome c oxidase subunit I (COI) gene, partial cds; mitochondrial	*Drosophila*	*hydei*	99.45%	OK037196.1
	*Drosophila hydei* isolate J86 cytochrome c oxidase subunit I (COI) gene, partial cds; mitochondrial	*Drosophila*	*hydei*	99.37%	KX275231.1
	*Drosophila hydei* isolate hydei54 cytochrome c oxidase subunit 1 (COI) gene, partial cds; mitochondrial	*Drosophila*	*hydei*	98.90%	MH142773.1

**FIGURE 3:**
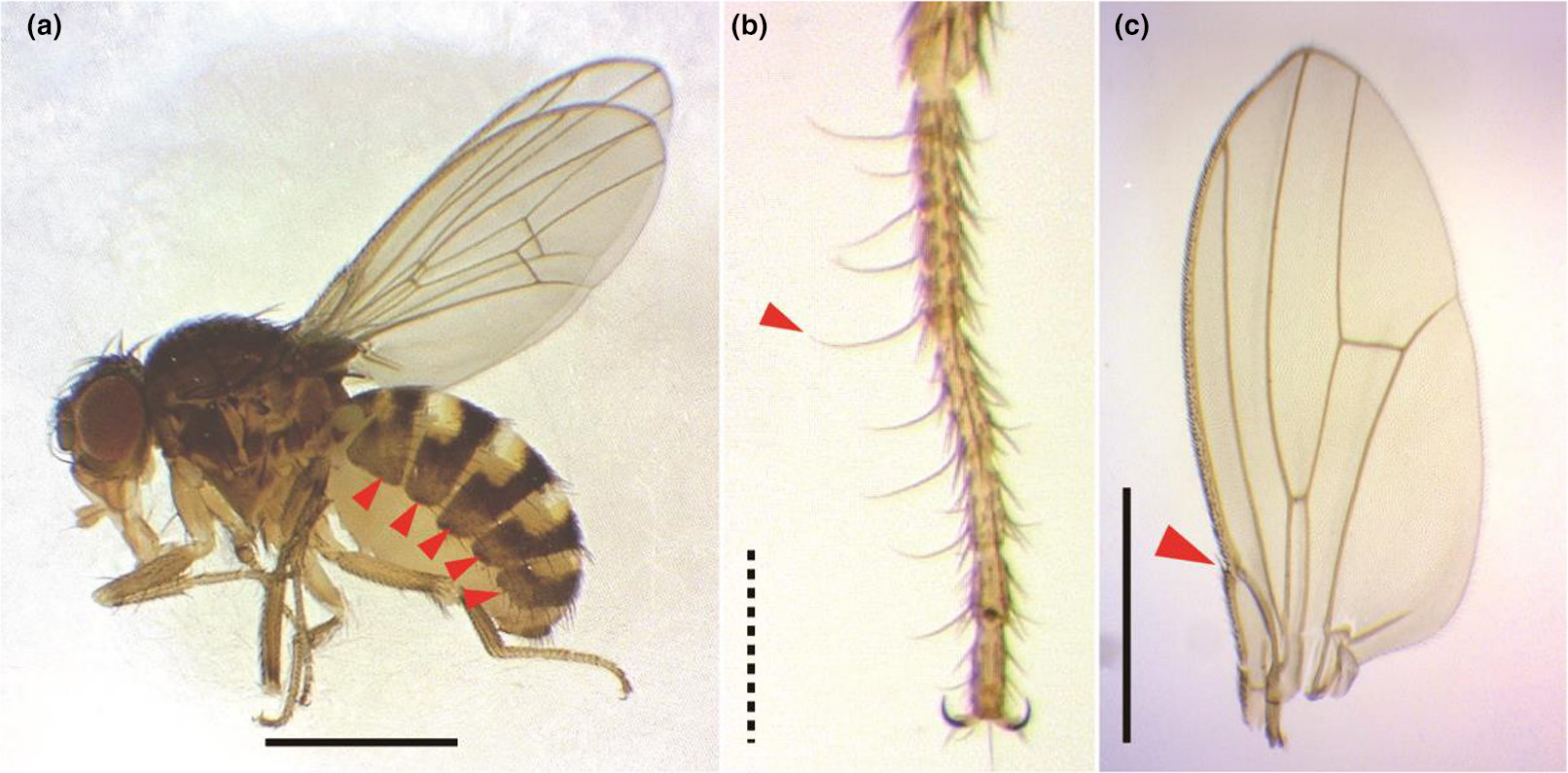
Diagnosis of *Torinido‐shoujoubae* (specimens from Wild Sky). (a) habitus (lateral view); (b) male fore tarsus (ventral view); (c) left wing (ventral view). Arrow heads indicate diffuse pale areas on posterolateral abdominal bands (a), prominent fine setae on male fore tarsus (b), and tip of subcostal break (c), respectively. Scale: solid line = 1 mm; dotted line = 0.2 mm.

**FIGURE 4:**
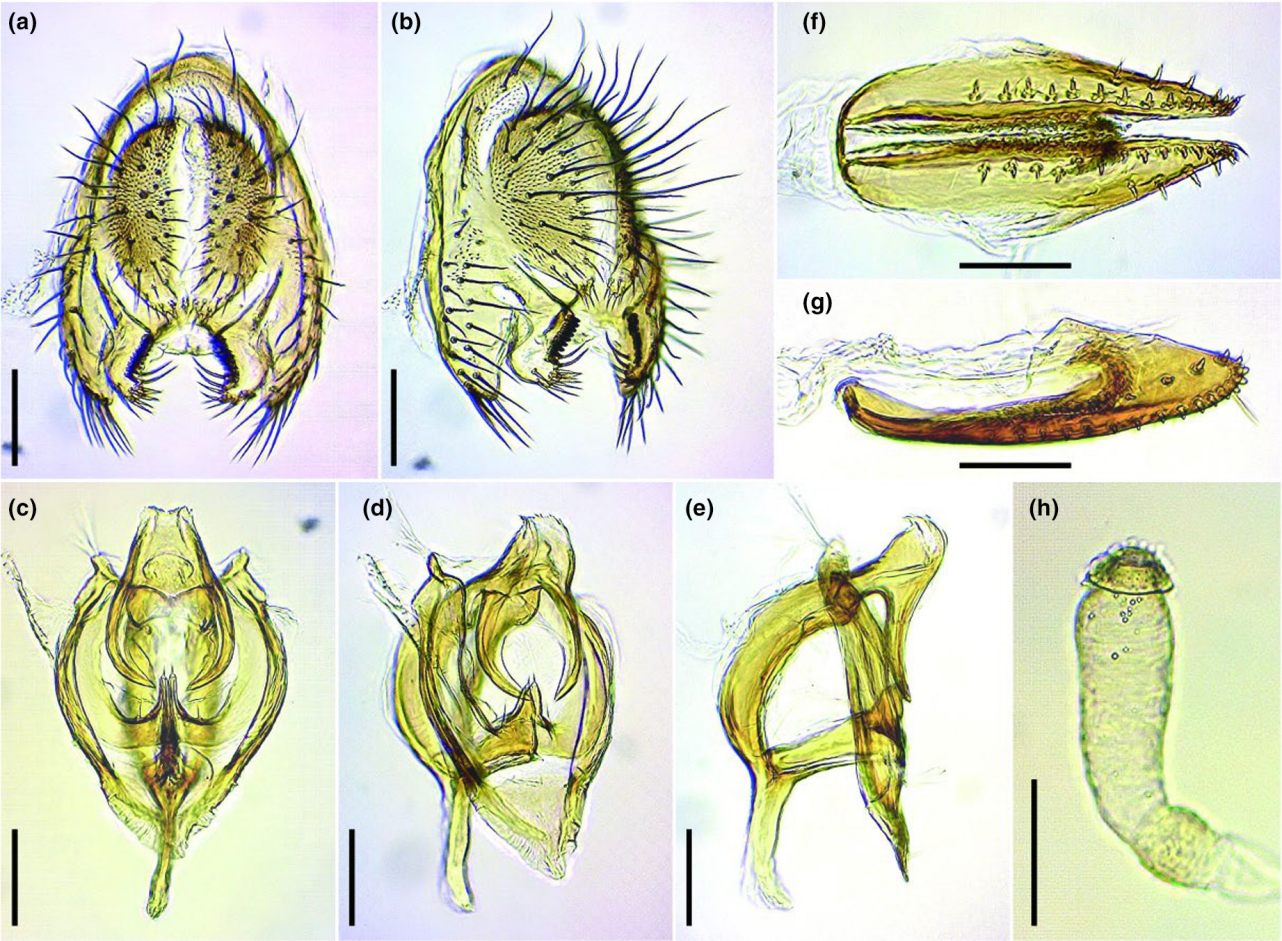
Male and female terminalia of *Torinido‐shoujoubae* (specimens from Wild Sky). (a, b) periphallic organs (caudal and caudolateral view, respectively); (c–e) phallic organs (ventral, ventrolateral, and lateral view, respectively); (f, g) oviscapt (ventral and lateral view, respectively); (h) spermatheca. Scale: solid line = 0.1 mm.

*Torinido‐shoujoubae* certainly belongs to *Drosophila repleta* species group (Sturtevant, 1921) in light of their morphological affinity to it, and identified as a member of this group, *D. hydei* because of their consistency with the diagnosis of *D. hydei* proposed in the original description (Sturtevant, 1921) and redescription (Bächli et al., 2004). The diagnosis of *D. hydei* were as follows (slightly modified from Bächli et al., 2004): lateral areas of posterolateral abdominal bands with diffuse pale areas only (Figure 3a); male fore tarsi with prominent, fine setae on inner side (Figure 3b); tip of subcostal break not darkened (Figure 3c). The general morphological features and the structures of male and female terminalia examined in the present study (Figure 4) were identical to those of *D. hydei* described in the literatures (e.g., Bächli et al., 2004; Hsu, 1949; Miller et al., 2017; Sturtevant, 1921).

The study revealed that all *Torinido*‐*shoujoubae* examined were *D. hydei*, through both molecular and morphological techniques. Flightless flies used in studies outside Japan are commonly recognized as *D*. *hydei* or its alternative choice, *D*. *melanogaster* (Vucic‐Pestic et al., 2010; Webber et al., 2010). The exact reason for the name of the fly to be altered remains unknown but has been hypothesized in the previous study with the morphological analysis that it was originated from a miscommunication of handwritten Japanese characters (Arai et al., 2020). The clarification from both morphological and molecular analysis within this study, there is a need for using the recognized scientific name of *D. hydei* or its Japanese name of *Kasuri*‐*shoujoubae*, instead of the name, *Torinido*‐*shoujoubae* with unknown origins. This would ensure both the reliability of the products in the Japanese market as well as the affirmation from the researchers using them. Miscommunications regarding names or details are not confined to any particular language. Confirmation of the scientific name of the used species enables the replicability of research which is a global process involving individuals from various background and languages. For instance, different feeds can affect the behaviors and expressions of the spiders with their web productions (Blamires et al., 2015; Craig et al., 2000). In conclusion, the importance of accurate scientific nomenclature cannot be overstated, as it not only ensures the reliability of research findings but also facilitates global collaboration and replicability in scientific endeavors across linguistic and cultural boundaries.

## AUTHOR CONTRIBUTIONS

**Koh Nakagawa:** Formal analysis (lead); investigation (lead); methodology (lead); project administration (lead); resources (lead); validation (equal); writing – original draft (equal); writing – review and editing (equal). **Kaoru Ogino:** Data curation (equal); formal analysis (equal); investigation (supporting); visualization (lead); writing – original draft (lead); writing – review and editing (equal). **Takehiro K. Katoh:** Formal analysis (equal); methodology (equal); supervision (equal); validation (equal); writing – original draft (equal) **Nobuaki Kono:** Funding acquisition (lead); methodology (equal); project administration (lead); validation (lead); writing – original draft (equal); writing – review and editing (equal).

## CONFLICT OF INTEREST STATEMENT

The authors have no competing interests to disclose.

## Data Availability

The COI sequences produced were deposited in GenBank under the accession numbers PP663876, PP663875, PP663877, and PP663878.
