# Correction to “Species Identification of Livefood Flightless Fly (Torinido‐Shoujoubae) Through DNA Barcoding”

**DOI:** 10.1002/ece3.70864

**Published:** 2025-01-19

Nakagawa, K., Ogino, K., Katoh, T. K., & Kono, N. Species identification of livefood flightless fly (Torinido‐shoujoubae) through DNA barcoding. *Ecology and Evolution*, 14(7):e11622.

In the “INTRODUCTION” section, paragraph 3, the phrase “In a previous study” was not clearly specified. The phrase should be corrected to “In a previous study (Araye et al., 2020)”.

In the “RESULTS AND DISCUSSION” section, the citation “Arai et al., 2020” contained an incorrect author name. The correct citation is “Araye et al., 2020”.

The reference “Arai, K., Onuma, M., & Sawamura, K. (2020). Torinido‐shoujoubae No Shoutai [identity of Torinido‐shoujoubae]. *Insect DNA Research Newsletter*, 32, 39–40.” contained an incorrect author name. The correct reference is “Araye, Q., Onuma, M., & Sawamura, K. (2020). Torinido‐shoujoubae No Shoutai [identity of Torinido‐shoujoubae]. *Insect DNA Research Newsletter*, 32, 39–40.”

We apologize for this error.

